# Insomnia and Its Therapeutics

**Published:** 1890-06

**Authors:** 


*Insomnia and its Therapeutics.* By A. W. MACFARLANE, M.D. Pp. 366. London: H. K. LEWIS. 1890.

This is a charmingly-written work, showing evidence of wide and close thinking, and a very varied and extensive knowledge of medicine. The author has evidently read much and to good purpose. The work is thorough in the fullest sense—in the ground it covers, in the exhaustiveness of its treatment, and in the soundness and resource of its therapeutics. It is the best monograph on the subject of sleeplessness which we have seen.

